# Supplementary material for: The incidence of acute respiratory infection in Indonesian infants and association with vitamin D deficiency
Source: PLoS One. 2021 Mar 23;16(3):e0248722. doi: 10.1371/journal.pone.0248722 (PMC7987198; doi:10.1371/journal.pone.0248722)
Supplement: S1 File — (PDF) [file pone.0248722.s001.pdf]

QUESTIONNAIRE 1 (Age 0-4 weeks)

The Burden Of Pneumonia In Indonesia Children And The Association With Vitamin D Deficiency: The Indonesian Pneumonia And Vitamin D Study (I-PADS)

Study ID ☐☐ / ☐☐☐☐ / ☐ - ☐☐☐ / I-PADS

<u>Instruction:</u> The study team will verbally administer the questionnaire by interviewing parent. Circle or complete answers that apply

1. Today's date ☐☐ / ☐☐ / ☐☐☐☐ (dd/mm/yyyy)
2. Study staff's initial ☐☐
3. The person answering the question is the 1. Mother 2. Father

Demographic details

4. Child's name ____________________

5. Parents' detail

   5.1 Parents' name Mother____________________
   Father____________________

   5.2 Address ____________________
   ____

   5.3 Phone number Mother ☐☐☐☐☐☐☐☐☐☐☐☐☐
   Father ☐☐☐☐☐☐☐☐☐☐☐☐☐

   5.4 Mother's Date of Birth : ☐☐ / ☐☐ / ☐☐☐☐ (dd/mm/yyyy)

   5.5 Parents' education (please fill out based on the code)
   code: 1: Middle school or less 2: High school
   3: University 4: Other

   Mother ☐ Father ☐

   5.6 Parents' employment status (please fill out based on the code)
   code: 1: Not working 2: student 3: Employee 4: Laborer
   5: House wife 6: Retired 7: Other

   Mother ☐ Father ☐

6. Alternative contact
   Name ____
   Relationship with the participant ____
   Address ____
   ____

   Phone number ☐☐☐☐☐☐☐☐☐☐☐☐

7. Map
   (Please draw us your home location from the PHC/ city landmarks)

SOCIO ECONOMIC STATUS

8. Total household monthly income per month. (Please circle the correct response)

   1. <Rp 500.000,00
   2. Rp 500.000-Rp1.000.000
   3. Rp 1.000.000-Rp2.500.000
   4. Rp 2.500.000-Rp 5.000.000
   5. >Rp 5.000.000

9. Are you covered by health insurance? (Please circle the correct response)

   1. Yes
   2. No

RISK FACTORS

10. How many people live in the household where the baby lives (not including baby)? (Please complete the boxes)

    ☐☐ people

11. Do any family members currently smoke (in the last 12 months)? (Please put the number that applies)

    11.1 Mother ☐
    11.2 Father ☐
    11.3 Other family member ☐
    (living in the same roof with baby)

    1. Not at all
    2. Yes, daily
    3. Yes, at least weekly
    4. Yes at least monthly

12. If yes, on average how many cigarettes do you smoke? (Please put the number that applies)

    12.1 Mother ☐
    12.2 Father ☐
    12.3 Other family member ☐
    (living in the same roof with baby)

    1. < 12 units
    2. 12-24 units
    3. > 24 units

13. The main material for cooking. (Please circle the correct response)

    1. Electric
    2. Gas
    3. Kerosene
    4. Wood
    5. Charcoal
    6. Other

The following questions will provide us with information about the mother's pregnancy history. Please kindly circle that apply or fill out the boxes

14. What was the gestational age of the baby at birth?

    ☐☐weeks + ☐☐ days

15. How many previous pregnancies has the mother had (excluding the most recent). (Please circle the correct response, if the answer is more than 3 please put the number that applies in the boxes)

    1. none 2. one 3. Two 4. Three 5. >three, mention ☐☐

16. How many other living children does mother have? (Please circle the correct response, if the answer is more than 3 please put the number that applies in the boxes)

    1. None 2. One 3. Two 4. Three 5. >three, mention ☐☐

17. Did the mother take any calcium supplements during her pregnancy? (Please circle the correct response)

1. No   2. Yes, but not regularly   3. Yes, regularly

18. Did mother take any other nutritional supplements? (Please circle the correct response)
1. Yes   2. No

18.1 If yes, please give detail of product name and dosage:
Brand ____
Dosage ____

18.2 If yes, please give detail of product name and dosage:
Brand ____
Dosage ____

19. What vitamin D containing foods did the mother take during her pregnancy? (Please circle the correct response and mention the frequency)

Foods	Yes	No	How many times (in average per week)
Milk and alternatives	1	2	_________ Times/week
Red Meat and alternatives	1	2	Times/week _________
Fish	1	2	_________ Times/week
Egg yolk	1	2	_________ Times/week

Sun exposure

20. How often is the mother being outside of the home during pregnancy? (Please put the number that applies) (silakan isikan nomor sesuai jawaban)

20.1 **On weekdays**   1. Yes, __________times/week   2. No
20.2 **On weekend**   1. Yes,__________ times/week   2. No

21. At what time of the day did the mother usually take go outside the house during pregnancy?(Please put the number that applies)

No	Hour	On Weekdays	On Weekend
21.1	Before 10.00 am		
21.2	Between 10.00 am – 16.00 pm		
21.3	After 16.00 pm		

22. On average, how many hours was the mother staying outside during pregnancy? (Please put the number that applies)

22.1 On a weekday ☐   22.2On a weekend ☐
1. Less than 15 min   4. 1 to 2 hours   5. 4 to 5 hours
2. 15 min to 30 min   5.2 to 3 hours   6. 5 to 6 hours

3. 31 min to 1 hour

23. How often did mother use sun protector during pregnancy? (Please circle the correct response)

23.1 Sun screen usage

No	Usage	Weekdays	Weekend
1	Always		
2	< 50% of the time		
3	≥ 50% of the time		
4	Not at all		

23.2 Sunglasses usage

No	Usage	Weekdays	Weekend
1	Always		
2	< 50% of the time		
3	≥ 50% of the time		
4	Not at all		

23.3 Umbrella usage

No	Usage	Weekdays	Weekend
1	Always		
2	< 50% of the time		
3	≥ 50% of the time		
4	Not at all		

24. What was the mother wearing during outdoor activities during pregnancy? (Please circle the correct response)

24.1 Arms exposed

No	Arms	On weekday	On weekend
1	Not exposed at all		
2	< 50% time exposed		
3	≥ 50% time exposed		
4	Always exposed		

24.2 Lower extremity exposed (thigh to heel)

No	Lower extremity	On weekday	On weekend
1	Not exposed at all		
2	< 50% time exposed		
3	≥ 50% time exposed		
4	Always exposed		

25. Does the mother intend to exclusively breastfeed her baby? (Please circle the correct response)

1. Yes 2. No

26. Who is the primary caregiver of the baby? (Please circle the correct response)

1. Mother 2. Father 3. Other family member 4. Babysitter

5. Others, please mention ______________

KUESIONER 1 (Usia 0-4 minggu)

Beban Penyakit Pneumonia pada Anak-anak di Indonesia dan Hubungan dengan Defisiensi Vitamin D: Penelitian Indonesian Pneumonia and Vitamin D (I-PAD)

ID Peserta ☐☐ / ☐☐☐☐ / ☐ - ☐☐☐ / I-PADS

<u>Petunjuk:</u> Tim peneliti akan mewawancarai secara verbal kepada orangtua. Lingkari atau lengkapi pertanyaan yang tersedia

1. Tanggal hari ini ☐☐ / ☐☐ / ☐☐☐☐ (dd/mm/yyyy)
2. Inisial pewawancara ☐☐
3. Pertanyaan dijawab oleh 1. Ibu 2. Bapak

Data Demografi

4. Nama Bayi ____

5. Data Orangtua
   - 5.1 Nama Orangtua Ibu :____
     Bapak :____
   - 5.2 Alamat :____
     ____
   - 5.3 Nomor telepon Ibu ☐☐☐☐☐☐☐☐☐☐☐☐☐
     Bapak ☐☐☐☐☐☐☐☐☐☐☐☐☐
   - 5.4 Tanggal lahir ibu : ☐☐ / ☐☐ / ☐☐☐☐ (dd/mm/yyyy)
   - 5.5 Pendidikan orangtua (isilah kotak berdasarkan kode yang tersedia)
     kode: 1: SMP atau dibawahnya 2: SMA atau sederajat
     3: Universitas atau sederajat 4: Lain-lain

     Ibu ☐ Bapak ☐
   - 5.6 Status pekerjaan orangtua (isilah kotak berdasarkan kode yang tersedia)
     kode: 1: Tidak berkerja 2: Siswa 3: Pegawai 4: Buruh
     5: IRT 6: Pensiunan 7: lain-lain

     Ibu ☐ Bapak ☐

6. Kontak Alternatif
   Nama : ______________________________
   Hubungan dengan peserta: ______________________________
   Alamat : ______________________________
   ____

   Nomor Telepon | | | | | | | | | | | | |

7. Peta
   (gambar peta lokasi rumah Anda dari puskesmas/BPM)

STATUS SOSIAL EKONOMI

8. Berapa total pendapatan perbulan?(lingkari jawaban yang sesuai)
    1. <Rp 500.000,00
    2. Rp 500.000-Rp1.000.000
    3. Rp 1.000.000-Rp2.500.000
    4. Rp 2.500.000-Rp 5.000.000
    5. >Rp 5.000.000

9. Apakah Anda memiliki asuransi kesehatan? (lingkari jawaban yang sesuai)
    1. Ya
    2. Tidak

FAKTOR RISIKO

10. Berapa banyak orang yang tinggal satu rumah bersama bayi (tidak termasuk bayi)? (Lengkapi kotak yang tersedia)
    ☐☐ orang

11. Apakah ada anggota keluarga yang merokok (dalam 1 tahun)? (isilah berdasarkan kode nomor yang tersedia)
    - 11.1 Ibu ☐
    - 11.2 Bapak ☐
    - 11.3 Anggota keluarga lain ☐ *(yang tinggal satu rumah dengan anak)*

    1. Tidak semua
    2. Ya, setiap hari
    3. Ya, minimal setiap minggu
    4. Ya, minimal 1 bulan

12. Jika iya, berapa rata-rata jumlah rokok yang dikonsumsi? (isilah berdasarkan kode nomor yang tersedia)
    - 12.1 Ibu ☐
    - 12.2 Bapak ☐
    - 12.3 Anggota keluarga lain ☐ *(yang tinggal satu rumah dengan anak)*

    1. ≤ 12 batang
    2. 13-24 batang
    3. > 24 batang

13. Bahan bakar utama untuk memasak. (lingkari jawaban yang sesuai)
    1. Listrik
    2. Gas
    3. Minyak tanah
    4. Kayu
    5. Arang
    6. Lain-lain

Pertanyaan berikut akan memberikan informasi tentang riwayat kehamilan ibu. Silakan lingkari atau isi kotak yang tersedia sesuai jawaban.

14. Berapa umur kehamilan ibu?
    ☐☐ minggu + ☐☐ hari

15. Berapa jumlah kehamilan terdahulu (tidak termasuk kehamilan sekarang)? (silakan lingkari jawaban yang sesuai, jika jawaban lebih dari 3 isilah jawaban pada kotak yang tersedia)

    1. Tidak ada 2. Satu 3. Dua 4. Tiga 5. >Tiga, sebutkan ☐☐

16. Berapa banyak jumlah anak ibu yang hidup? (silakan lingkari jawaban yang sesuai, jika jawaban lebih dari 3 isilah jawaban pada kotak yang tersedia)

    1. Tidak ada 2. Satu 3. Dua 4. Tiga 5. >Tiga, sebutkan ☐☐

17. Apakah ibu mengonsumsi suplemen kalsium selama kehamilan? (silakan lingkari jawaban yang sesuai)

    1. Tidak 2. Ya, tetapi tidak rutin 3.Ya, rutin

18. Apakah ibu mengonsumsi suplemen nutrisi yang lain? (silakan lingkari jawaban yang sesuai)
    1. Ya 2. Tidak

    18.1 Jika iya, sebutkan merk/nama produk dan dosisnya:
    Merk ____
    Dosis ____

    18.2 Jika iya, sebutkan merk/nama produk dan dosisnya:
    Merk ____
    Dosis ____

19. Makanan apa yang mengandung vitamin D yang dikonsumsi ibu selama kehamilan? (silakan lingkari jawaban yang sesuai dan sebutkan jadwalnya)

Makanan	Ya	Tidak	Rata-rata konsumsi dalam 1 minggu
Susu	1	2	__________ kali/minggu
Daging merah	1	2	__________ kali/minggu
Ikan	1	2	__________ kali/minggu
Kuning telur	1	2	__________ kali/minggu

Paparan sinar matahari

20. Seberapa sering ibu berada di luar rumah selama kehamilan? (silakan isikan nomor sesuai jawaban)
    20.1 **Pada hari kerja** 1. Ya, __________kali/minggu 2. Tidak
    20.2 **Pada akhir pekan** 1. Ya,__________ kali/minggu 2. Tidak

21. Kapan biasanya ibu berada di luar rumah selama kehamilan? (silakan beri tanda centang pada jawaban yang sesuai)

No	Jam	Pada hari Kerja	Pada akhir pekan
21.1	Sebelum jam 10.00		
21.2	Antara jam 10.00 – 16.00		
21.3	Setelah jam 16.00		

22. Rata-rata berapa lama ibu biasa berada di luar rumah di bawah matahari langsung selama kehamilan? (silakan isikan nomor sesuai jawaban)
    22.1 **Pada hari kerja** ☐ 22.2 **Pada akhir pekan** ☐

    1. kurang dari 15 menit
    2. 15 - 30 menit
    3. 31 menit - 1 jam
    4. 2 jam
    5. 3 jam
    6. 4 jam
    7. 5 jam

23. Seberapa sering ibu memakai pelindung matahari selama kehamilan? (silakan beri tanda centang pada jawaban yang sesuai)

23.1 Penggunaan tabir surya

No	Penggunaan	Pada hari Kerja	Pada akhir pekan
1	Selalu menggunakan		
2	Menggunakan < 50% waktu terpapar		
3	Menggunakan ≥ 50% waktu terpapar		
4	Tidak menggunakan sama sekali		

23.2 Penggunaan kacamata hitam

No	Penggunaan	Pada hari Kerja	Pada akhir pekan
1	Selalu menggunakan		
2	Menggunakan < 50% waktu terpapar		
3	Menggunakan ≥ 50% waktu terpapar		
4	Tidak menggunakan sama sekali		

23.3 Penggunaan payung

No	Penggunaan	Pada hari Kerja	Pada akhir pekan
1	Selalu menggunakan		
2	Menggunakan < 50% waktu terpapar		
3	Menggunakan ≥ 50% waktu terpapar		
4	Tidak menggunakan sama sekali		

24. Bagaimanakah kebiasaan berpakaian ibu ketika berada di luar rumah selama kehamilan? (silakan beri tanda centang pada jawaban yang sesuai)

24.1 Terekspos bagian lengan

No	Bagian Lengan	Pada hari Kerja	Pada akhir pekan
1	Tidak terpapar sama sekali		
2	< 50% waktu terpapar		
3	≥ 50% waktu terpapar		
4	Selalu terpapar		

24.2 Terekspos bagian ekstremitas bawah (paha hingga tumit)

No	Bagian ekstremitas bawah	Pada hari Kerja	Pada akhir pekan
1	Tidak terpapar sama sekali		
2	< 50% waktu terpapar		
3	≥ 50% waktu terpapar		
4	Selalu terpapar		

25. Apakah ibu berniat untuk menyusui bayinya secara eksklusif? (silakan lingkari jawaban yang sesuai)
    1. Ya   2. Tidak

26. Siapakah yang menjadi pengasuh utama bayi? (silakan lingkari jawaban yang sesuai)
    1. Ibu   2. Ayah   3. Keluarga lain   4. Pengasuh bayi/Babysitter
    5. Lainnya, sebutkan ______________

IPADS COMMUNITY : EPISODES OF ACUTE RESPIRATORY ILLNESSES (ARI)

ID ☐☐/☐☐☐☐/☐-☐☐☐ MR : ☐☐☐☐☐☐ Form No : ☐☐ Baby Name: ______________________________ DOB ☐☐/☐☐/☐☐
☐☐

		Episode 1	Episode 2	Episode 3	Episode 4	Episode 5	Episode 6	Episode 7	Episode 8	Episode 9	Episode 10
Age in months											
Diagnosis: 1. Doctor diagnosed pneumonia/LRTI<br>2. URTI<br>3. Other dx, mention		1<br>2<br>3	1<br>2<br>3	1<br>2<br>3	1<br>2<br>3	1<br>2<br>3	1<br>2<br>3	1<br>2<br>3	1<br>2<br>3	1<br>2<br>3	1<br>2<br>3
Source of information:<br>1. Parents self-report<br>2. Medical record/doctor/nurse<br>3. Both		1<br>2<br>3	1<br>2<br>3	1<br>2<br>3	1<br>2<br>3	1<br>2<br>3	1<br>2<br>3	1<br>2<br>3	1<br>2<br>3	1<br>2<br>3	1<br>2<br>3
Start date		/ /	/ /	/ /	/ /	/ /	/ /	/ /	/ /	/ /	/ /
End date		/ /	/ /	/ /	/ /	/ /	/ /	/ /	/ /	/ /	/ /
Symptom present											
1. Fever<br>2. Cough<br>3. Runny nose<br>4. Dyspnea/Fast breathing<br>5. Chest Indrawing<br>6. Wheeze	7. Diarrhea/Vomit<br>8. Danger signs<br>a. Unable to drink<br>b. Vomit everything<br>c. Unconscious<br>d. Stridor<br>e. Cyanosis	1 7<br>2 8a<br>3 8b<br>4 8c<br>5 8d<br>6 8e<br>9	1 7<br>2 8a<br>3 8b<br>4 8c<br>5 8d<br>6 8e<br>9	1 7<br>2 8a<br>3 8b<br>4 8c<br>5 8d<br>6 8e<br>9	1 7<br>2 8a<br>3 8b<br>4 8c<br>5 8d<br>6 8e<br>9	1 7<br>2 8a<br>3 8b<br>4 8c<br>5 8d<br>6 8e<br>9	1 7<br>2 8a<br>3 8b<br>4 8c<br>5 8d<br>6 8e<br>9	1 7<br>2 8a<br>3 8b<br>4 8c<br>5 8d<br>6 8e<br>9	1 7<br>2 8a<br>3 8b<br>4 8c<br>5 8d<br>6 8e<br>9	1 7<br>2 8a<br>3 8b<br>4 8c<br>5 8d<br>6 8e<br>9	1 7<br>2 8a<br>3 8b<br>4 8c<br>5 8d<br>6 8e<br>9
	9. Other, mention. ...										
Antibiotic	1. Yes 0. No										
a. Cotrimoxazole<br>b. Erythromycin<br>c. Cefotaxime<br>d. Penicillin<br>e. Ampicillin	f. Amoxicillin<br>g. Gentamycine<br>h. Amoxicillin clavulanat<br>i. Ceftriaxone<br>j. others, mention,	a f<br>b g<br>c h<br>d i<br>e j	a f<br>b g<br>c h<br>d i<br>e j	a f<br>b g<br>c h<br>d i<br>e j	a f<br>b g<br>c h<br>d i<br>e j	a f<br>b g<br>c h<br>d i<br>e j	a f<br>b g<br>c h<br>d i<br>e j	a f<br>b g<br>c h<br>d i<br>e j	a f<br>b g<br>c h<br>d i<br>e j	a f<br>b g<br>c h<br>d i<br>e j	a f<br>b g<br>c h<br>d i<br>e j
	Mention other antibiotics										
Nebulized: B2 agonist	1. Yes 0. No										
Steroid	1. Yes 0. No										
Cough Medicine,<br>Name__________________	1. Yes 0. No										
Oral Salbutamol	1. Yes 0. No										
CTM	1. Yes 0. No										
Outcome:											
1. Self limiting (no treatment)<br>2. Outpatient, remission<br>3. Hospitalisation, cured<br>4. Died		1<br>2<br>3<br>4	1<br>2<br>3<br>4	1<br>2<br>3<br>4	1<br>2<br>3<br>4	1<br>2<br>3<br>4	1<br>2<br>3<br>4	1<br>2<br>3<br>4	1<br>2<br>3<br>4	1<br>2<br>3<br>4	1<br>2<br>3<br>4
Notes											

THE UNIVERSITY OF MELBOURNE Murdoch Childrens Research Institute

IPADS - KOMUNITAS: EPISODE INFEKSI SAURAN PERNAPASAN AKUT (ISPA)

ID ☐☐/☐☐☐☐/☐-☐☐☐ No. RM : ☐☐☐☐☐☐☐ No. formulir: ☐☐ Nama bayi: ______________________________ Tanggal lahir ☐☐/☐☐/☐☐☐☐

			Episode 1		Episode 2		Episode 3		Episode 4		Episode 5		Episode 6		Episode 7		Episode 8		Episode 9		Episode 10	
Usia dalam bulan																						
Diagnosis:																						
1. Diagnosis pneumonia/ISPA bag. bawah oleh dokter<br>2. ISPA bag. atas<br>3. Diagnosis lainnya, sebutkan			1<br>2<br>3		1<br>2<br>3		1<br>2<br>3		1<br>2<br>3		1<br>2<br>3		1<br>2<br>3		1<br>2<br>3		1<br>2<br>3		1<br>2<br>3		1<br>2<br>3	
Sumber informasi:																						
1. Laporan orangtua<br>2. Rekam medis/ dokter/ perawat<br>3. Keduanya			1<br>2<br>3		1<br>2<br>3		1<br>2<br>3		1<br>2<br>3		1<br>2<br>3		1<br>2<br>3		1<br>2<br>3		1<br>2<br>3		1<br>2<br>3		1<br>2<br>3	
Tanggal mulai			/ /		/ /		/ /		/ /		/ /		/ /		/ /		/ /		/ /		/ /	
Tanggal berakhir			/ /		/ /		/ /		/ /		/ /		/ /		/ /		/ /		/ /		/ /	
Gejala yang muncul																						
1. Demam<br>2. Batuk<br>3. Hidung meler<br>4. *Dispnu*/Napas cepat<br>5. Tarikan dinding dada bawah ke dalam<br>6. Mengi<br>7. Diare/ muntah	8. Tanda bahaya<br>a. Tidak bisa menyusu<br>b. Memuntahkan semua yang diminum<br>c. Penurunan kesadaran<br>*d. Stridor*<br>e. Sianosis		1<br>2<br>3<br>4<br>5<br>6<br>7	8a<br>8b<br>8c<br>8d<br>8e<br>9	1<br>2<br>3<br>4<br>5<br>6<br>7	8a<br>8b<br>8c<br>8d<br>8e<br>9	1<br>2<br>3<br>4<br>5<br>6<br>7	8a<br>8b<br>8c<br>8d<br>8e<br>9	1<br>2<br>3<br>4<br>5<br>6<br>7	8a<br>8b<br>8c<br>8d<br>8e<br>9	1<br>2<br>3<br>4<br>5<br>6<br>7	8a<br>8b<br>8c<br>8d<br>8e<br>9	1<br>2<br>3<br>4<br>5<br>6<br>7	8a<br>8b<br>8c<br>8d<br>8e<br>9	1<br>2<br>3<br>4<br>5<br>6<br>7	8a<br>8b<br>8c<br>8d<br>8e<br>9	1<br>2<br>3<br>4<br>5<br>6<br>7	8a<br>8b<br>8c<br>8d<br>8e<br>9	1<br>2<br>3<br>4<br>5<br>6<br>7	8a<br>8b<br>8c<br>8d<br>8e<br>9	1<br>2<br>3<br>4<br>5<br>6<br>7	8a<br>8b<br>8c<br>8d<br>8e<br>9
	9. Lainnya, sebutkan ...																					
Antibiotik	1. Ya	0. Tidak																				
a. Cotrimoxazole<br>*b. Erythromycin*<br>*c. Cefotaxime*<br>*d. Penicillin*<br>*e. Ampicillin*	*f. Amoxicillin*<br>*g. Gentamycine*<br>*h. Amoxicillin clavulanat*<br>*i. Ceftriaxone*<br>j. Lainnya, sebutkan		a<br>b<br>c<br>d<br>e	f<br>g<br>h<br>i<br>j	a<br>b<br>c<br>d<br>e	f<br>g<br>h<br>i<br>j	a<br>b<br>c<br>d<br>e	f<br>g<br>h<br>i<br>j	a<br>b<br>c<br>d<br>e	f<br>g<br>h<br>i<br>j	a<br>b<br>c<br>d<br>e	f<br>g<br>h<br>i<br>j	a<br>b<br>c<br>d<br>e	f<br>g<br>h<br>i<br>j	a<br>b<br>c<br>d<br>e	f<br>g<br>h<br>i<br>j	a<br>b<br>c<br>d<br>e	f<br>g<br>h<br>i<br>j	a<br>b<br>c<br>d<br>e	f<br>g<br>h<br>i<br>j	a<br>b<br>c<br>d<br>e	f<br>g<br>h<br>i<br>j
	Antibiotik lainnya																					
Nebulisasi: *B2 agonist*	1. Ya	0. Tidak																				
Steroid	1. Ya	0. Tidak																				
Obat batuk,<br>Nama__________________	1. Ya	0. Tidak																				
Salbutamol per oral	1. Ya	0. Tidak																				
CTM	1. Ya	0. Tidak																				
Hasil akhir:																						
1. Sembuh tanpa pemberian obat<br>2. Rawat jalan, sembuh<br>3. Rawat inap, sembuh<br>4. Meninggal			1<br>2<br>3<br>4		1<br>2<br>3<br>4		1<br>2<br>3<br>4		1<br>2<br>3<br>4		1<br>2<br>3<br>4		1<br>2<br>3<br>4		1<br>2<br>3<br>4		1<br>2<br>3<br>4		1<br>2<br>3<br>4		1<br>2<br>3<br>4	
Catatan tambahan																						

IPADS COMMUNITY: GROWTH, FEEDING AND SUN BEHAVIOR

ID ☐☐/☐☐☐☐/☐-☐☐☐ MR : ☐☐☐☐☐☐☐ Baby Name: ________________________________ DOB ☐☐/☐☐/☐☐☐☐

Variables			1	2	3	4	5	6	7	8	9	10	11	12
FU date			/ /	/ /	/ /	/ /	/ /	/ /	/ /		/ /	/ /		/ /
Current weight (in gr)														
Current length (in cms)														
Arm circumference (in cms)														
Feeding														
1. Breastfeed<br>2. Formula milk<br>3. Solid food<br>4. Mineral water or water-based drinks<br>5. Fruit juice and sugar			1<br>2<br>3<br>4<br>5	1<br>2<br>3<br>4<br>5	1<br>2<br>3<br>4<br>5	1<br>2<br>3<br>4<br>5	1<br>2<br>3<br>4<br>5	1<br>2<br>3<br>4<br>5	1<br>2<br>3<br>4<br>5		1<br>2<br>3<br>4<br>5	1<br>2<br>3<br>4<br>5		1<br>2<br>3<br>4<br>5
Vitamin D food intake														
Milk	0. None<br>1. Once/ month<br>2. Once/ week<br>3. 2-3/week<br>4. 4-5/week<br>5. Everyday													
Red meats														
Fish														
Egg yolk														
Do you usually take your baby outside the house under the sunlight?														
1. Yes (if yes, please mention how frequent in a weekdays/end)<br>0. No		Weekdays												
		Weekend												
Take the baby outside the house under the sunlight														
1. Before 10.00 am<br>2. Between 10.00 am- 4.00 pm<br>3. After 4.00 pm		Weekdays												
		Weekend												
When do you usually take your baby outside the house under the sunlight?														
1. < 15 minutes<br>2. 15-30 minutes<br>3. 31 minutes – 1hour<br>4. 2 hours	5. 3 hours<br>6. 4 hours<br>7. 5 hours	Weekdays												
		Weekend												
Baby wear during the time he/she was exposed to the sun:														
Sun Exposure:<br>1. Not exposed at all<br>2. <50% time exposed<br>3. ≥ 50% time exposed<br>4. Always exposed	Arms exposed	Weekdays												
		Weekend												
	Lower extremity exposed (thigh to heel)	Weekdays												
		Weekend												
Sun Protection:<br>1. Always<br>2. <50% of the time<br>3. ≥ 50% of the time<br>4. Not at all	Sunscreen usage	Weekdays												
		Weekend												
	Sunglasses usage	Weekdays												
		Weekend												
	Umbrella usage	Weekdays												
		Weekend												
Baby go to a daycare	1. Yes, at what age (in months) 2. No													
Baby's primary caregiver	1. Mother 3. Other, mention<br>2. Father													

(Column group 1–12 headed: Age in months)

IPADS - KOMUNITAS: PERTUMBUHAN, ASUPAN DAN PERILAKU BERJEMUR

ID ☐☐/☐☐☐☐/☐-☐☐☐ No. RM : ☐☐☐☐☐☐☐ Nama Bayi : ____________________ Tgl. Lahir ☐☐/☐☐/☐☐☐☐

Variabel			Usia dalam bulan 1	2	3	4	5	6	7	8	9	10	11	12
Tanggal wawancara			/ /	/ /	/ /	/ /	/ /	/ /	/ /		/ /	/ /		/ /
Berat badan saat wawancara (gram)														
Pandang badan saat wawancara (cm)														
Lingkar lengan atas saat wawancara (cm)														
Asupan														
1. Air Susu Ibu<br>2. Susu formula<br>3. Makanan padat<br>4. Air mineral atau minumam lainnya<br>5. Jus buah and gula			1<br>2<br>3<br>4<br>5	1<br>2<br>3<br>4<br>5	1<br>2<br>3<br>4<br>5	1<br>2<br>3<br>4<br>5	1<br>2<br>3<br>4<br>5	1<br>2<br>3<br>4<br>5	1<br>2<br>3<br>4<br>5		1<br>2<br>3<br>4<br>5	1<br>2<br>3<br>4<br>5		1<br>2<br>3<br>4<br>5
Asupan makanan sumber vitamin D														
Susu	0. Tidak pernah<br>1. 1x/bulan<br>2. 1x/minggu<br>3. 2-3x/minggu<br>4. 4-5x/minggu<br>5. Setiap hari													
Daging merah														
Ikan														
Kuning telur														
Apakah Anda biasanya mengajak bayi Anda keluar rumah dan terpapar sinar matahari?														
1. Ya (jika Ya, seberapa sering dilakukan pada hari kerja atau akhir pekan?)<br>0. Tidak		Hari kerja												
		Akhir pekan												
Kapankah Anda biasanya mengajak bayi Anda keluar rumah dan terpapar sinar matahari?														
1. Sebelum pukul 10.00 WIB<br>2. Antara pukul 10.00 dan 16.00 WIB<br>3. Setelah 4.00 WIB		Hari kerja												
		Akhir pekan												
Berapa lama Anda biasanya mengajak bayi Anda keluar rumah dan terpapar sinar matahari?														
1. < 15 menit<br>2. 15-30 menit<br>3. 31 menit – 1 jam<br>4. 2 jam	5. 3 jam<br>6. 4 jam<br>7. 5 jam	Hari kerja												
		Akhir pekan												
Seberapa sering bagian lengan dan kaki bayi Anda terpapar sinar matahari pada saat keluar rumah?														
Frekuensi paparan sinar matahari:<br>1. Tidak pernah terpapar<br>2. <50% dari waktu terpapar<br>3. ≥ 50% dari waktu terpapar<br>4. Selalu terpapar	Bagian lengan	Hari kerja												
		Akhir pekan												
	Bagian kaki (paha hingga tumit/ujung kaki)	Hari kerja												
		Akhir pekan												
Bagaimanakah upaya Anda melindungi bayi Anda dari paparan sinar matahari pada saat keluar rumah?														
Upaya melindungi tubuh bayi dari sinar matahari:<br>1. Selalu<br>2. <50% dari waktu terpapar<br>3. ≥ 50% dari waktu terpapar<br>4. Tidak pernah sama sekali	Penggunaan tabir surya	Hari kerja												
		Akhir pekan												
	Penggunaan kacamata hitam	Hari kerja												
		Akhir pekan												
	Penggunaan payung	Hari kerja												
		Akhir pekan												
Apakah bayi Anda mengikuti PAUD/ Tempat penitipan anak?	1. Jika Ya, sebutkan usia dalam bulan<br>2. Tidak													
Pengasuh utama bayi	1. Ibu 2. Bapak 3. Lainnya, sebutkan													

Version 5. Dated 29 December 2015

Series number : STC - ☐☐☐
(completed by resarch assistant)

STANDARDISED CLINICAL RECORD

Date of visit : ☐☐ / ☐☐ / ☐☐☐☐ dd/mm/yyyy
Child's name :
Participant's ID : ☐☐ / ☐☐☐☐/ ☐ - ☐☐☐ / I-PADS
DOB : ☐☐ / ☐☐ / ☐☐☐☐ dd/mm/yyyy

No. MR : ☐☐☐☐☐☐☐☐☐☐☐
Site : **PHC……………………./ Midwife clinic………………………..**
Age : ☐☐ **months/years old***(circle appropriate answer)*

Doctor's name :
Signature :

Instruction: Please complete this form and leave it in child's medical record in primary health care center or midwife clinic!

Main Symptom(s)	Danger Sign	Body Weight	Examination	Classification	Therapy
☐ Fever ☐☐☐ day(s) ago<br>☐ Runny nose ☐☐☐ day(s) ago<br>☐ Cough ☐☐☐ day(s) ago<br>☐ Fast breathing* ☐☐☐ day(s) ago<br>☐ Wheeze ☐☐☐ day(s) ago<br>☐ Diarrhea ☐☐☐ day(s) ago<br>☐ Others,_________ ☐☐☐ day(s) ago<br>____________	☐ None<br>☐ Unable to drink/feed<br>☐ Vomit everything<br>☐ Lethargic/unconscious<br>☐ Stridor<br>☐ Cyanosis<br>☐ Underweight<br>☐ Others<br>_____________	☐☐☐,☐ Kgs<br>**Length**<br>☐☐☐ cms	**Respiratory rate in 60 seconds:**<br>**1st count*:** ☐☐☐/min<br>**2nd count (if increases)*** ☐☐☐/min<br>**Chest indrawing:**<br>☐ Yes<br>☐ No	☐ Not pneumonia<br>☐ Pneumonia<br>☐ Severe Pneumonia<br>☐ Others,<br>___________	☐ Cough medicine, mention___________<br>_______________<br>☐ Antibiotic<br>_______________<br>☐ Oral salbutamol<br>☐ Inhalation salbutamol<br>☐ Steroid<br>☐ Zinc<br>☐ ORS<br>☐ Others,__________
**Follow up plan:** ☐ Outpatient ☐ Hospitalised ☐ Referred		**Place for follow up plan:**		**Research Assistant's name:**<br>**Signature:**	

***Fast breathing criteria:** ≥ 60 bpm for < 2 months age; ≥ 50 bpm for 2 – 11 months age; ≥ 40 bpm for 12 – 59 months age

Versi 5. Tanggal 29 Desember 2015

No. Seri : CKS - ☐☐☐
(hanya diisi oleh tim peneliti)

LEMBAR CATATAN KLINIS STANDAR

Tanggal kunjungan : ☐☐ / ☐☐ / ☐☐☐☐ dd/mm/yyyy
Nama Anak :
ID peserta : ☐☐ / ☐☐☐☐/ ☐ - ☐☐☐ / I-PADS
Tanggal lahir anak : ☐☐ / ☐☐ / ☐☐☐☐ dd/mm/yyyy

No. RM : ☐☐☐☐☐☐☐☐☐☐☐
Site : **Puskesmas……………………./ BPM………………………..**
Usia : ☐☐ **bulan/tahun** *(coret salah satu)*

Nama Dokter :
Tanda tangan :

<u>Instruksi</u>: Mohon lengkapi form ini dan <u>lampirkan pada rekam medis anak</u> di puskesmas/ BPM

Gejala utama	Tanda bahaya	Berat badan	Pemeriksaan	Klasifikasi	Terapi
☐ Demam, ☐☐☐ hari yang lalu<br>☐ Pilek ☐☐☐ hari yang lalu<br>☐ Batuk ☐☐☐ hari yang lalu<br>☐ Napas Cepat* ☐☐☐ hari yang lalu<br>☐ Mengi ☐☐☐ hari yang lalu<br>☐ Diare ☐☐☐ hari yang lalu<br>☐ Lainnya,_______ ☐☐☐ hari yang lalu<br>_______________	☐ Tidak ada<br>☐ Tidak bisa menyusu/ makan-minum<br>☐ Memuntahkan apapun<br>☐ Lemah atau tidak sadarkan diri<br>☐ Stridor dalam kondisi tenang<br>☐ Sianosis<br>☐ Gizi Buruk<br>☐ Lain-lain<br>_____________	☐☐,☐ Kg<br>**Panjang Badan**<br>☐☐☐ cm	**Laju Napas/ RR dalam 1 menit penuh:**<br>**Hitungan 1*:** ☐☐☐/min<br>**Hitungan 2 (jika meningkat)*** ☐☐☐/min<br>**Tarikan dinding dada ke dalam:**<br>☐ Ya<br>☐ Tidak	☐ Bukan pneumonia<br>☐ Pneumonia<br>☐ Pneumonia berat<br>☐ Lainnya,<br>___________	☐ Obat batuk, sebutkan<br>_________________<br>☐ Antibiotik<br>_________________<br>☐ Salbutamol Oral<br>☐ Inhalation salbutamol<br>☐ Steroid<br>☐ Zinc<br>☐ ORS<br>☐ Lainnya,__________
**Tindak lanjut:** ☐ Rawat Jalan ☐ Rawat Inap ☐ Rujuk			**Tempat tindak lanjut:**	**Inisial tim PRO:**<br>**Tanda tangan tim PRO:**	

Admin *checklist* : Catatan klinis ☐ Terisi lengkap ☐ sudah tercatat pada log catatan klinis ☐ disimpan dalam file peserta

Paraf Admin :_________________

***Kriteria napas cepat:** ≥ 60 npm untuk usia < 2 bulan; ≥ 50 npm untuk usia 2 – 11 bulan; ≥ 40 npm untuk usia 12 – 59 bulan
