# Supplementary material for: The incidence of acute respiratory infection in Indonesian infants and association with vitamin D deficiency
Source: PLoS One. 2021 Mar 23;16(3):e0248722. doi: 10.1371/journal.pone.0248722 (PMC7987198; doi:10.1371/journal.pone.0248722)
Supplement: Attachment — Submitted filename: Review Comments.docx [file pone.0248722.s002.docx]

Oktaria et al. tried to investigate the vitamin-D levels of the ARI affected infants and the possible correlation of this vitamin with the incidence of ARI. Eventually they concluded that Vitamin D deficiency was associated with the incidence of ARI but not of pneumonia in the first year of life but adequate maternal sun exposure during pregnancy was associated with a trend to less frequent ARI and pneumonia in Indonesian infants.

My observations-

1. Here the authors mentioned that similar prospective cohort community-based cohort studies that measure actual pneumonia incidence are limited. However, if they can compare or discuss results from any other Indonesian or South Asian studies about ARI in infants especially with regards to immunization/breastfeeding practices/socioeconomic status, it would make the research paper even better and add more credibility to the results.
2. Despite the great efforts of the authors, the data obtained in this study lacks innovation, it is recommended that the paper be submitted to the local journals.
