# Supplementary material for: The incidence of acute respiratory infection in Indonesian infants and association with vitamin D deficiency
Source: PLoS One. 2021 Mar 23;16(3):e0248722. doi: 10.1371/journal.pone.0248722 (PMC7987198; doi:10.1371/journal.pone.0248722)
Supplement: Attachment — Submitted filename: review_Oktaria_et_al_PONE-D-20-33592_24_Dec_2020.docx [file pone.0248722.s003.docx]

Dear PLOS ONE Editors and Oktaria et al.,

**RE: “The incidence of acute respiratory infection in Indonesian infants and association with vitamin D deficiency”**

**PONE-D-20-33592**

Many thanks for the opportunity to review this paper. We found the study interesting, important and overall well-performed.

Please see comments below:

**Abstract**

1. Lines 62-63: Conclusion: Minimising vitamin D deficiency at birth such as by supplementation or safe sun exposure 63 has the potential to reduce ARI incidence in infants in this setting.” >Supplementation of mothers during pregnancy?

**Introduction**

2. The introduction is informative, presents some background knowledge on the subject and literature. However, I would suggest providing some more specific information on the Indonesian population e.g.:
   a. What is the prevalence of ARI in Indonesia, how many infants diagnosed each year, mortality rate, etc.
   b. What are some known risk factors of ARIs and how does this relate to the Indonesian population?
   c. Are ARIs the leading cause of death in Indonesian children? What about different age groups?
   d. Also, some additional background information is required regarding pneumonia in children.
   e. What about prevalence of vitamin D deficiency in Indonesian children?

This will help to articulate the rationale and the objectives of the study more clearly.

3. Line 109-111: discussion of limited evidence currently reads a bit confusing, would recommend some re-wording.
4. Lines 85-91 >After line 86 I would suggest having a sentence to introduce the reader that ARI can affect either upper respiratory system or lower respiratory system and then go into details of each URTI, LRTI, and pneumonia more specifically.
5. Line 95 >Please provide more details regarding the Haemophilus influenzae type b vaccine in the introduction e.g. When was it implemented? Does it prevent pneumonia?
6. Page 5 lines 106-108 “Subclinical vitamin D deficiency (serum vitamin D <50 nmol/L) is far more common and has…” And Line 180: We defined 180 vitamin D deficiency as a serum 25-hydroxyvitamin D level of <50nmol/L.
   a. It would be good to provide a reference for these statements.
   b. Add “far more common in infants” if this is this what the sentence implies.
7. Page 6 lines 108-111 “However, evidence of a causal relationship is inconclusive and limited studies of vitamin D supplementation have not yet demonstrated benefit for prevention or treatment outcomes [9, 15]”
   a. I assume the abovementioned limited studies are in children, or do they include adults as well?
   b. Could rephrase to: A limited number of studies on vitamin D supplementation in children with ARI have been published to date and evidence of a convincing association with prevention or treatment of outcomes is still lacking.
8. Line 129 Study procedures: Any exclusion criteria that should be mentioned? Both parents were interviewed or only mother?
9. Lines 146-147 “ if the participants reported cough for more than three days to obtain the respiratory rate” >Do the participants imply the infants? And whenever the word participants is used I would suggest to use infants for consistency.
10. Line 150 according to WHO guidelines > Reference?

**Methods**

11. Line 144: It would be good to have a description of what constituted “respiratory symptoms”. It is a bit unclear if a classification of “ARI non-pneumonia episode” required a home visit to be considered for the study (e.g. cough over 3 days?) and how these episodes were categorized.

**Statistical analysis**

12. Which confounders were considered for the Cox Regression, please provide more details.
13. I can see that certain sensitivity analyses were conducted e.g. using a lower cut-off for vitamin D (level of <25) but these are not measured in methods. It would be good to mention these in the statistical analysis.
14. Was the proportional hazards assumption for Cox proportional hazards models tested?
15. I don’t see any apparent risk of immortal person time according to classification of exposures and outcomes.
16. Line 171-172: It is possible that seasonality impacts maternal sun exposure, unclear if this is accounted for in the calculation of cumulative composite estimate UVR (Wierzejska, R., Jarosz, M., Sawicki, W., Bachanek, M. and Siuba-Strzelińska, M., 2017. Vitamin D concentration in maternal and umbilical cord blood by season. International journal of environmental research and public health, 14(10), p.1121.)
17. Data are not available to reviewers. Please upload these and deposit somewhere accessible.
18. Analyses scripts are not available either. Please upload these as well and deposit somewhere accessible.

**Results**

19. “Between December 2015 and December 2017, we recruited 422 participants with 95% 210 (400/422) of infants retained for the full twelve months of follow-up.”
    a. Participant is mothers or infants?
    b. Any important information for those lost to follow up?
20. Please show plots as well as tables.
21. Lines 224-225 The demographics characteristics of those with vitamin D samples at birth and six months of 225 age were representative of the entire cohort (Table 1).
    a. Specify “those”, infants?
    b. Change participants to infants in Table 1

**Discussion**

22. Line 349-350: A major potential confounder exists in the assessment of maternal sun exposure – since this is likely to be higher in more rural areas and infections may be more prevalent in urban ones. Was there any measure available for urbanicity? Household crowding was considered but it is not reflective of wider environment.
23. Line 361: Vitamin D supplementation is discussed, was there any data collection on maternal use of supplements?
24. It seems a bit odd to me that deficiency according to cord blood would be so high, is this normal? I couldn’t find similar figures through a quick literature search.

Many thanks to the authors and congratulations on conducting a long and non-trivial study. We hope comments are useful and look forward to your reply.
